# Women's Experience of the Consent Process to Planned Caesarean Section and Its Surgical Risk: A Qualitative Study

**DOI:** 10.1111/1471-0528.18049

**Published:** 2024-12-25

**Authors:** Malathy Nithiyananthan, Jacqueline Nicholls, Melissa Whitten, Katherine Maslowski, Anne Lanceley

**Affiliations:** ^1^ UCL Medical School, Medical School Building University College London London UK; ^2^ Department of Reproductive Health, Faculty of Population Health Sciences Medical School Building, EGA Institute for Women's Health, University College London London UK; ^3^ Women's Health Division, Elizabeth Garrett Anderson Wing University College London Hospital NHS Foundation Trust London UK; ^4^ EGA Institute for Women's Health University College London London UK; ^5^ Department of Women's Cancer, Faculty of Population Health Sciences Medical School Building, EGA Institute for Women's Health, University College London London UK

**Keywords:** consent practice, elective caesarean section, informed consent, PPH, surgical risk

## Abstract

**Objective:**

To explore how women appreciated the risks discussed within the consent process for planned caesarean section (CS).

**Design:**

Exploratory qualitative interview study.

**Setting:**

NHS Teaching Hospital in Central London.

**Population:**

Women over the age of 18, English speaking, scheduled for a planned CS.

**Methods:**

Semi‐structured interviews were conducted before and after a woman's CS. Eighteen women were recruited and interviewed prior to undergoing CS and 12 of these were interviewed following CS. Interviews were audio‐recorded, transcribed and thematically analysed.

**Main Outcome Measures:**

Themes generated from analysis of interviews exploring the experiences of women consenting to CS and specifically their awareness of postpartum haemorrhage (PPH), hysterectomy, organ damage and risk of placental abnormalities in future pregnancies.

**Results:**

Two broad themes and four subthemes were identified (1) Untimely provision of risk information: (a) superficial risk discussions during the antenatal period and full risk disclosure on the day of surgery and (b) incompleteness absent or sparse risk disclosure prior to making the decision to undergo the CS, where women were unaware of specific risks and (2) Emotional overload: (a) fear of risks and (b) fear that a CS will be denied to them—women's cognitive response and notably their emotional response to their situation limited their understanding of risks disclosed.

**Conclusion:**

The consent process for planned CS was found to lack appropriate and full risk disclosure. Risk disclosure was ill‐timed or deficient in facilitating women's understanding of risks reflecting a consent process which does not meet legal and professional standards of informed consent.

## 1 Introduction

Around 8% of all births in England are completed via planned Caesarean section (CS), with a steady rise in the rate of CS over the last few decades [1]. Planned or elective CS is a surgical foetal delivery, decided in advance of labour, involving an incision through a woman's abdomen and uterus [2]. Planned CS can be medically indicated such as in cases of placenta praevia and multiple pregnancies or can be at the request of the pregnant individual [3]. While there is little evidence that a CS is safer for babies, reports of long‐term implications for mothers are increasing [1]. Complications of CS include infection, organ damage, thromboembolic complications and risk to future pregnancies such as uterine rupture, infertility and placental abnormalities [4]. CS deliveries also carry an increased risk of postpartum haemorrhage (PPH) compared to other modes of delivery, affecting around 1 in 100 women [4, 5]. Severe PPH can require intervention including life‐saving emergency hysterectomy for 1 in 670 women [6], and blood transfusions. Royal College of Obstetricians and Gynaecology (RCOG) guidelines include a template consent form (Table 1) [7]. Obtaining informed consent for women prior to surgery is a non‐negotiable legal and professional requirement intended to foster women's choice making [8, 9, 10]. A consent requirement is the provision of adequate time [7] to allow for a woman‐professional dialogue which supports a thorough understanding of presented information enabling individuals to make informed decisions [10]. The requirements for ‘dialogue’ are emphasised in the landmark UK legal case of Montgomery v Lanarkshire as needing to ‘ensure the patient understands the anticipated benefits and risks of the proposed treatment. The ruling states “[the doctors] role will only be performed effectively if the information provided is comprehensible’ and that ‘the doctor's duty is not fulfilled by bombarding the patient with technical information she cannot grasp’ para 90 [11]. The case of Montgomery [11], echoing an earlier Australian case of Rogers v Whittaker [12], and subsequent cases have established a two‐limbed test to judge risk disclosure both objectively and subjectively, underpinning that ‘material’ risk disclosure for a ‘reasonable’ and ‘particular’ person facilitates informed consent. Further legal cases have endorsed the requirements of materiality for risk disclosure [13, 14].

**TABLE 1:** RCOG consent form.

Consent form for planned caesarean birth 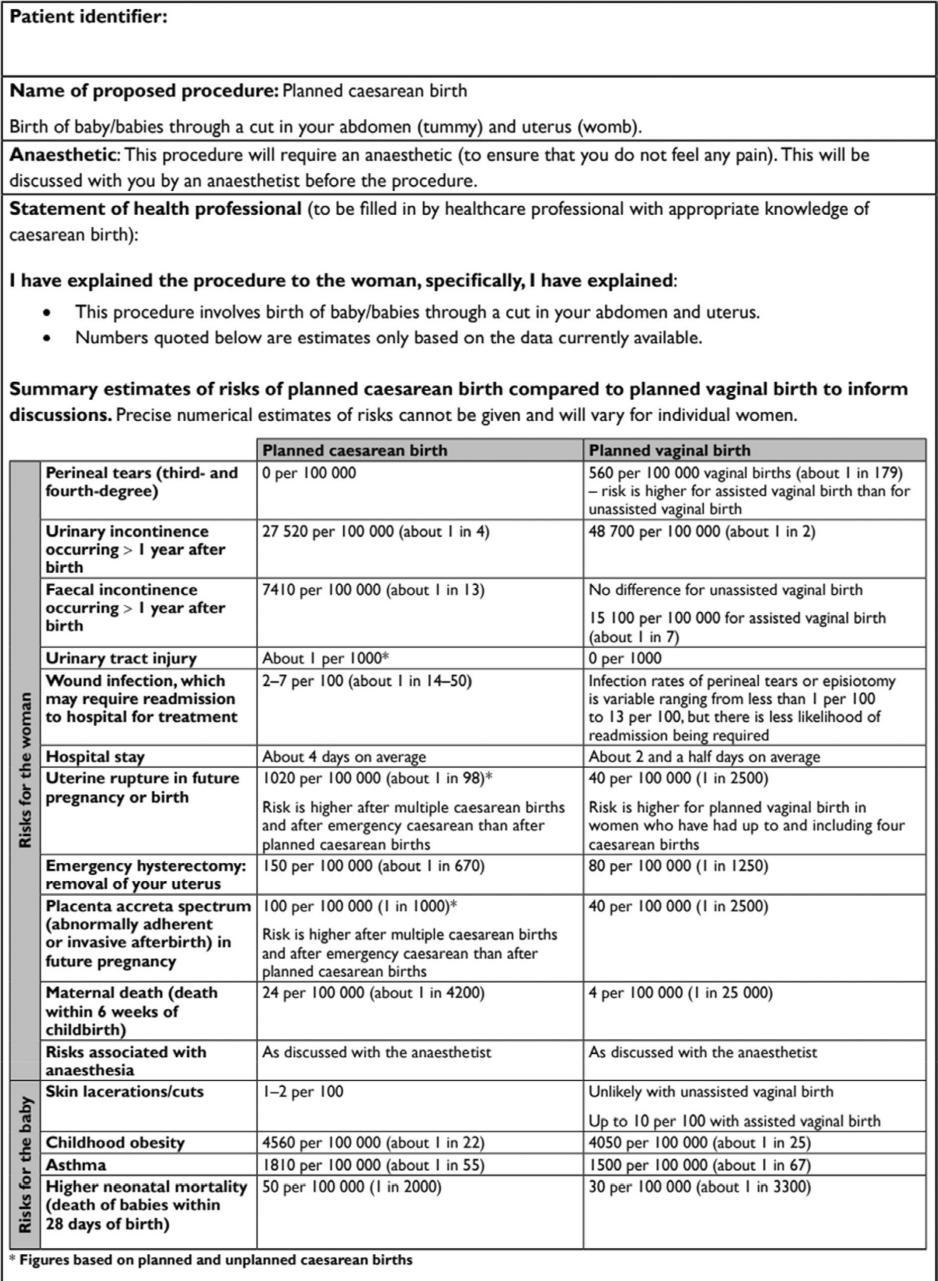
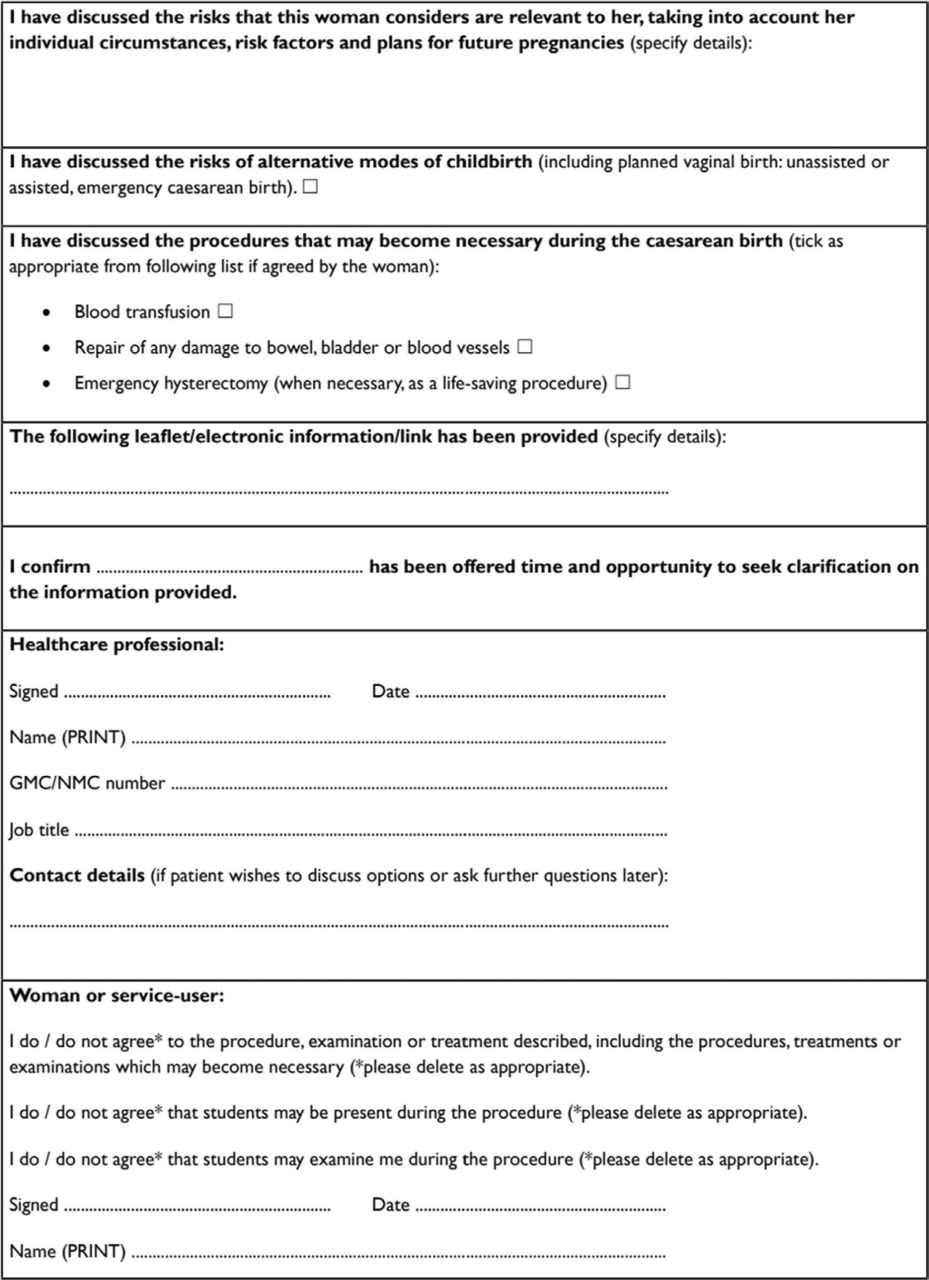

Existing legal requirements [11] and previous work [15, 16] highlight the challenges involved in fostering complex discussions of information and risk disclosure in ways which facilitate decision‐making, informed consent and expression of choice. The literature on consent for planned CS is very limited. There are currently no UK‐based studies assessing women's understanding of risk during the consent process for planned CS. A study conducted at a tertiary centre in Ethiopia revealed that there was inadequate informed consent across planned and emergency CS [17].

This study aimed to explore woman's experiences of consent to planned CS with specific reference to discussion of risk.

## 2 Methods

### 2.1 Study Design

This exploratory longitudinal qualitative study used semi‐structured interviews conducted both before and after CS in each woman. The design was chosen as it enabled us to explore women's perspectives of their consent experience over time [18]. The study design, analysis and interpretation were framed by medico‐legal theory concerned with the practical problems of law [18].

The study was developed as a result of conversations between the Human Rights Charity, Birthrights and the research team. Ethical approval was granted by the Health Research Authority (REC 17/YH/0212) as part of a wider research initiative exploring women's choices and consent in health care.

The study is reported with reference to the consolidated criteria for reporting qualitative research (COREQ) [19].

### 2.2 Participants

Participants were identified by a convenience sampling method. All individuals undergoing a planned CS at an NHS Teaching Hospital in central London were scheduled for a pre‐assessment appointment 2–3 days before their operation, at which point, they were invited by a member of their clinical team to take part in the study. Those who were interested were introduced to the researcher in a private setting and were given additional details about the study including a Participant Information Sheet. Following an opportunity to ask questions and discuss any concerns, individuals who wished to participate gave written consent. Individuals included in the study were undergoing their first or subsequent planned CS, understood spoken and written English and were over the age of 18.

### 2.3 Data Collection

The interview topic guide was developed and tested before use in a similar population by members of the research team experienced in qualitative women's healthcare research (AL and JN). It was subsequently reviewed by the wider study team (MN, KM and MW). Members of the study team (KM, AL, MW and JN) identify as white females and are all healthcare professionals. AL is a nurse, JN a physiotherapist, MW is a Consultant Obstetrician, and KM is a doctor with obstetric experience. JN is also a trained solicitor with expertise in health law. MN a female medical student of Sri Lankan Tamil heritage, conducted the interviews with women whom she had not met prior to the research commencing.

The one‐on‐one interviews were conducted in private either on the antenatal ward or via telephone for the pre‐ and post‐section interviews respectively. Participants were interviewed at a time convenient to them and could choose the mode of their post‐section interview, that is, in person or by telephone. This was important for the follow‐up interviews when participants were adapting to caring for their new baby. They were informed that the interview could be paused or stopped at any time to protect their well‐being and autonomy.

An interview schedule including a set of secondary probe questions was developed from a schedule previously piloted and tested in a similar population by study researchers AL and JN [20]. The interview guide (Table 2) was finalised following discussions with the research team (MN, KM, AL and JN), and clinical professionals to gauge how to efficiently explore participant's understanding, expression of choice and information provision. For example, the question ‘were risks discussed with you’ in the interview guide was refined to ‘do you recall any risks and benefits of a CS, when were they first discussed, and how were they explained to you?’ The guide questions were designed to address the CS consent process with a particular focus on depth of risk disclosure, specifically addressing awareness of the risk of PPH, hysterectomy, organ (bowel/bladder) damage and risk to future pregnancies. These risks are commonly included in the consent form for planned CS which participants would sign prior to the operation. Previous similar qualitative studies in maternity care guided our initial sample size estimate [20] which was finally determined through reaching data saturation across both interviews and at the level of analysis. Data saturation was iteratively monitored during recruitment and recruitment ceased in the absence of new emerging codes and themes across the pre and post CS interviews.

**TABLE 2:** Interview topic guide.

**Purpose**
What do you understand by consent?
How does it relate to your view of you making decisions about your care?
**Initial approach**
How was the issue of consent raised with you?
**Who**
How were you prepared when you were asked for your consent? (were you given any preliminary information?)
What do you feel your role was in the process?
How was the purpose of the consent process explained to you?
**What—Content**
What information was given to you in relation to the decision you were being asked to give your consent for?
Were you given any information sheets, websites or other sources of information?
What do you think are the important things to address when consenting a patient?
Were risks discussed with you? How were they explained to you?
Were benefits discussed with you? How were they explained to you?
**How—Process**
What difficulties, if any, did you experience when you were asked for your consent?
Did the doctor check your understanding when seeking consent?
Did you ask any questions?
If so did you feel satisfied by the answers you received?
What do you think is the purpose of the consent form?
In terms of the paperwork you were provided with: Were there any areas that you found confusing? Was it clear to you what you were giving your consent to? Were you asked for your consent in relation to hypothetical future possibilities (e.g., Can your partner use your gametes if you die?). How did you feel about this? How easy was it to make a decision about giving your consent?

Interviews were audio‐recorded and were subsequently transcribed by a UCL‐approved confidential transcription agency. The transcripts were checked against the original audio‐recordings to confirm accuracy.

Demographic data including age, ethnicity, number of previous CS and reason for CS were obtained from participants' medical records. Socio‐economic status (NS‐SEC Class) was also recorded [21].

### 2.4 Data Analysis

All transcripts were thematically analysed by hand by MN following Braun and Clarke's six step method [22]. Transcripts were read and re‐read to facilitate familiarisation with the data. The entire data set was systematically and iteratively examined by MN to identify recurring patterns, topics and experiences that were labelled into codes. These codes were organised and categorised into meaningful groups to systematically understand and interpret the dialogue. The codes were discussed and refined by the research team and synthesised into initial themes which were reviewed, and revised. Cross‐checking of codes and themes between members of the research team maintained analytical rigour.

In our analysis, we were alert to potential nuanced differences in the data obtained from in‐person and telephone data collection methods [23]. There was no discernible difference in the quality of the data and participants spoke candidly in all interviews. We were alert also to possible differences in response between women who had previously had a CS and those who had not. Throughout the research, we were also mindful of the influence of the research team member's gender, ethnicity, profession and values and assumptions of what constitutes high‐quality maternity care. There were inherent advantages and potential biases in our experiences, and we were cognisant of how these might play out in the interview dynamic and the interpretation of data [24].

## 3 Results

A total of 31 women were approached to take part in the study. Eighteen women consented to take part, with 13 declining due to either limited time availability before their CS or a disinterest in taking part. A 67% follow‐up rate was achieved with 12 participants completing both a pre‐ and post‐CS interview. Each pre‐ and post‐CS interview lasted up to 25 min. The information provided in the demographic Table 3 is not aligned to individual participants in the interests of patient confidentiality.

**TABLE 3:** Participant demographics.

Age (no. of women)	25–29	1
	30–34	5
	35–39	7
	40–44	5
	Asian Bangladeshi	1
	Asian other	2
Ethnicity (no. of women)	Mixed white and black African	1
	White British	9
	White other	5
Socio‐economic status (NS‐SEC) (avg. %)	Higher managerial, administrative and professional occupations	27.0%
	Never worked and long‐term unemployed	8.2%
No. of prev. CS (no. of women)	0	13
	1–2	5
Reason for CS (no. of women)	Medically indicated	8
	Maternally requested	10

Qualitative analysis revealed two broad themes.

1. Untimely provision of risk information

- Superficial risk discussion during antenatal period and full risk disclosure on the day of surgery
  Women were questioned about their discussions with HCPs regarding their CS, specifically, discussions including risk disclosure. Many of them described a superficial level of risk disclosure during the ante‐natal period, followed by complete risk disclosure on the day of surgery at which point written consent was taken. The back‐loading of information sharing immediately prior to the CS left women with insufficient time to fully understand the risks presented.

I am conscious of the fact that I will be coming in on Monday to be given what will be the whole lot of information on the day planned for the procedure. To then be able to digest that… (P12 Pre‐CS)

Most of the things which she explained to me [on the day of surgery] I didn't know [before]. (P3 Post‐CS)

The majority of women in their post‐CS interviews expressed the need for discussions to take place earlier rather than the ill‐timed risk disclosure on the day of surgery which was sometimes described as causing emotional distress. Similar to findings across the pre‐section data, difficulty in understanding risks presented on the day of surgery was also described in post‐CS interviews. Some women also stated that because the first mention of some risks did not occur until the day of surgery they felt unable to disagree with HCPs or withdraw consent as it was too late in the process.

I would have preferred [full risk disclosure within the written consent process] beforehand, so it's not too much to deal with on the day [of CS] because the day itself was overwhelming, it was a big day. (P7 Post‐CS)

I feel that [full risk disclosure within the written consent process] would be more beneficial being like a week before. Not an hour before […] an hour before the CS I'm not going to disagree with anything. (P8 Post‐CS)

[The written consent process] was the full package of stuff I was expecting at some point, I hadn't heard the full list up until that point […] it is overwhelming to have on the day itself […] a couple of hours before. (P12 Post‐CS)

b Absent or sparse risk disclosure prior to making the decision to undergo the CS.

Many women described limited discussions with HCPs regarding the risks of CS. There was a clear deficiency in information provided during the ante‐natal period, either partially or in its entirety.

For the majority of women, risks associated with CS, especially the risk of PPH, hysterectomy, organ damage and placental abnormalities in future pregnancies, were not disclosed either in full or in part prior to the day of surgery. In the pre‐CS interview, women were asked about what risks they could recall from conversations with HCPs throughout the antenatal period. Some women were unable to recall many risks and they reflected that this was because they had not been mentioned to them in the first instance.

There wasn't much discussion around some of those really quite key [risks] if this doesn't go quite to plan. (P2 Pre‐CS)

There's always a risk of complication. You need to be aware of it. Didn't really go into details about what they are. (P11 Pre‐CS)

Several women reported that there had been no discussion whatsoever prior to the day of surgery concerning the risks of a CS to ‘their’ health, all of which would be later mentioned and formally consented to on the day of surgery.

I don't even think we spoke about the risks and benefits yet. (P1 Pre‐CS).

It just occurs to me like okay this is major surgery for me, it feels like someone probably should have spoken to me about this from my point of view […] the risks and recovery. (P12 Pre‐CS)

No risks have really been discussed. (P17 Pre‐CS)

One woman specifically addressed in her post‐CS interview that she had limited understanding of all the risks presented even after antenatal discussions and the conversation in which formal written consent was taken.

It was more like you are signing your life away without really knowing anything. (P5 Post‐CS)

Women were specifically asked about whether PPH, hysterectomy, organ damage and any placental abnormalities in future pregnancies were mentioned to them prior to surgery. From the pre‐CS interviews, we found that 11 women were unaware of the risk of hysterectomy, nine were unaware of the risk of organ damage, seven were unaware of risk of PPH and seven were unaware of the risk of placental abnormalities in future pregnancies.

I think one thing I definitely hadn't considered is the risk of hysterectomy […] I haven't made the decision whether I've completed my family yet. And obviously that is kind of a major concern. (P6 Pre‐CS)

No, I don't remember any conversation about organ damage. […] Is there any? (P9 Pre‐CS)

Not yet [had the conversation about surgical risks of CS] […] Sorry, it's a little bit scary to have [hysterectomy mentioned] two days before you have [a CS] (P10 Pre‐CS)

2 Emotional Overload
  - Fear of CS risks

Around half of women reported experiencing emotions such as fear, anxiety and doubt at the time of being asked to make a decision which they considered impacted their ability to fully participate in a meaningful discussion of the risks. Fearfulness in hearing the risks of a CS was described, often coinciding with the bombardment of risk disclosure information on the day of surgery and when obtaining consent. Women were left struggling to process information contributing to limited understanding of these risks.

I don't remember all of [the risks] because I do take the advice and sometime get anxious myself speaking about it. So I don't fully digest everything […] I'm sure they would have explained [risks of CS]. (P9 Pre‐CS)

I know [HCPs] have to say all of the risks, but I think it can make it feel very disproportionate. At the end of that conversation [on risks] I was actually sick and had to put my legs up because I felt so faint from the sheer volume of horrors. […] It's just hard for it to sink in. (P14 Pre‐CS)

They did mention a whole list of things. Which to be honest it's quite scary and overwhelming especially before you're going in for the CS. (P9 Post‐CS)

b Fear that CS will be denied to them

The negative effect of emotions was described especially amongst the 10 women who requested a CS, in the absence of medical indication. They reported that the fear of being denied a CS compromised their participation in the discussion as they did not want to say anything which might prejudice their chances. This meant that they were sometimes reluctant to ask questions to clarify their understanding of the risks.

I didn't know whether the conversation was to assess whether I was eligible or not [for a CS], so I think I was more anxious around that which probably meant that I didn't retain as much information as I could have done […] I've been so hell bent that I'm having a caesarean that I was like yes, no its fine. (P16 Pre‐CS)

I think my worry was that I was going to be kind of rebuffed or told that there's no need for a C‐section […] I'm looking at [discussion on risk] from a slightly different angle. (P4 Pre‐CS)

I think maybe at the time [of being told the risks] my preoccupation was with actually whether they were going to be happy to do a CS […] whether I was actually thinking enough about what the risks were of the actual procedure, maybe I should have tried to go over that. (P6 Pre‐CS)

## 4 Discussion

### 4.1 Main Findings

This study found that women's experiences of the consent process for planned CS revealed minimal or defective communication of risk in a way which supported women in making a fully informed choice. Women's limited awareness and understanding of the surgical risks associated with CS raises serious questions regarding the adequacy of the consent process to meet legal and professional standards of informed consent. Our findings indicate that women's understanding of the surgical risks of planned CS is inadequately supported due to barriers to their understanding of risk. Barriers to understanding and therefore informed consent included either not receiving or at best receiving partial risk information in the antenatal period, and subsequently receiving a large quantity of information at the last minute of the consent process prior to surgery.

The need for adequate time in an optimal consent process seems to be more viable in a planned CS ante‐natal setting given discussions and decisions being made in advance of labour [7]. However, legally established principles for information disclosure on peri‐operative risks, outcomes, options of other treatments and follow‐up management were not proficiently discussed [25]. The requirement for risk disclosure despite it not having any bearing on a patient's decision to undertake the operation has been clarified in the Spencer v Hillingdon NHS trust case [26]. While it could be argued that the legal requirements are too demanding for the messy world of practice, our view is that their single purpose is to demand constant, iterative reflection on patient–professional interaction consistent with professional practice requirements [27].

Perfunctory discussions of risk prior to surgery and heavy reliance on risk‐dominated discussions on the day of surgery during the written consent process did not adequately meet the legal and professional standard of consent practice and patient‐centred care. The late and sometimes absent disclosure of information may be seen to undermine a woman's sense of agency and therefore genuine involvement in her care. This renders her vulnerable to implicit professional pressures [28] and in turn compromises her ability to make a genuinely autonomous decision.

Our study reveals that women's feelings of anxiety and distress during the consent process led to a state of emotional overload. In part, this was provoked by the fear that surgical risks would be realised along with the consequent emotional burden of making a high stakes decision regarding their pregnancy. This emotional overload was also observed in women requesting a CS in the absence of a clinical indication as they were too fearful of being denied a CS to engage in risk discussions. The additional deluge of information in an untimely matter exacerbated this emotional response and hindered women's ability to engage in a dialogue with their HCP concerning the risks of CS. This ultimately contributed to insufficient knowledge and comprehension of presented risks and therefore difficulties understanding a key element for decisional capacity and giving informed consent [29].

In this study, risk disclosure on the day of surgery when written consent was obtained for CS was a key period of information gathering for women. As existing literature suggests, the extensive listing of risks on the consent form to be signed may be used to ‘safeguard [the clinician] against the threat of litigation’ [30]. This consent form would be better utilised during the antenatal period as a supportive measure to facilitate informed consent and shared discussions on the risks of CS. However, the practice of presenting and ‘routinely demanding [a woman's] signature on a consent form’ para 90 [6] in the final hours leading up to surgery solely for legal purposes can lead to decisions being made under duress and in the absence of autonomy. Existing literature suggests that the consent form should be presented at the time an individual is listed for surgery to allow for ‘balanced discussion’ as a signature on a consent form without such discussion does not equate to informed consent [31]. One of the consequences of undue reliance on a late discussion of the risks of a CS was to render it even more difficult for a woman to withdraw her consent at such a late stage in the consent process. This raises concern in relation to the voluntariness of the consent given and therefore brings into doubt its validity.

We suggest that there is a strong case for clinicians to implement a consent process which explicitly fosters formal discussion of all risks in the ante‐natal period involving family members and the wider care team as appropriate. Such a process may include accumulative discussion of risks but would better support a woman's ability to make an autonomous decision [32]. Consent on the day of surgery should be used to reiterate previously discussed risks and verify that a woman still wishes to proceed rather than be used as a last‐minute opportunity to introduce previously undisclosed risks.

Existing evidence suggests that, in general, women disadvantaged by their ethnicity, disability or social circumstances have particularly poor consent experience [33, 34]. The issues that we have identified with English‐speaking women in our study indicate that the situation is likely worse for non‐English‐speaking women. Future work investigating minoritised women's experiences of a planned CS consent process is warranted. Our previous work in maternity care has identified HCP barriers to lawful consent. For example, HCPs on a labour ward experienced difficulty in judging appropriate risk disclosure and tended to view consent in terms of compliance [35]. A future study is needed to explore HCP perspectives on the consent process for planned CS, to determine the training needed to help professionals in deliberative dialogue with women to facilitate informed consent.

### 4.2 Interpretation

The lack of a two‐way deliberation to elicit and facilitate understanding of information appeared to contribute to an interaction based on an all‐knowing HCP and a passively receptive woman. Fostering understanding of information is reliant on providing adequate time and support for women to participate in balanced discussions once fears and anxieties have been addressed. Thorough comprehension of information and risks can therefore elicit autonomous decision‐making and informed consent [36]. Our results accord with previous research and conclude that risk communication—a component of informed consent—needs to be part of a broader dialogue to explore a patients' preferences [37, 38].

### 4.3 Strengths and Limitations

A key strength of this study was the conduct of interviews both before and after CS; a unique study design to encourage reflections from participants throughout the time between the two interviews when surgery and written consent would take place and after the delivery of their child. We achieved a good follow‐up rate of 67%.

A degree of recall bias is inherent in our design and participants' recollection of discussions of the surgical and other risks of CS may have been inaccurate as some of these took place several weeks or months before the research interview(s). An alternative study design could include observations of consultations and interviews with women at key points during the antenatal period. Whether the results from our study apply to other antenatal care services remains open although responses to this publication may strengthen such generalisability.

## 5 Conclusion

Information disclosure for planned CS needs to be relevant and significant to a particular person, expressed in a way that they can understand. From this study, it is evident that this cannot solely involve the stating of facts. Rather, it should consist of consent discussions that are timely, personal and responsive to the individual to ensure informed consent. Additional efforts to anticipate psychological burdens on decisional capacity would help to ensure risks presented are understood. Such changes in consent practice will ensure consent to CS is made by women who are appropriately informed and adequately prepared.

## Author Contributions

A.L. and J.N. designed the study and M.N. assisted with detailed planning. A.L. was the principal investigator. M.W. assisted with clinical access and study implementation. M.N. recruited participants and conducted interviews, after undertaking qualitative research training from A.L. and J.N., M.N. carried out data analysis, assisted by A.L. and J.N., M.N. wrote the manuscript, which all authors (M.W., K.M., J.N. and A.L.) participated in drafting, reviewing and final approval of.

## Ethics Statement

Ethical approval was granted by the UK Health Research Authority (REC Reference: 17/YH/0212) on 30/07/2017. Informed written consent was obtained from all participants before taking part in the study. Participants were aware of participation being voluntary and that they were able to withdraw from the study at any given point within the research process.

## Conflicts of Interest

The authors declare no conflicts of interest.

## Data Availability

Data that support the findings of this study are available upon request from the authors.
